# Improving Self-Awareness of Motor Symptoms in Patients With Parkinson’s Disease by Using Mindfulness – A Study Protocol for a Randomized Controlled Trial

**DOI:** 10.3389/fpsyg.2020.00743

**Published:** 2020-04-17

**Authors:** Timo Marcel Buchwitz, Franziska Maier, Andrea Greuel, Carsten Eggers

**Affiliations:** ^1^Department of Neurology, University Hospital Marburg, Marburg, Germany; ^2^Department of Psychiatry, University Hospital Cologne, Medical Faculty, Cologne, Germany; ^3^Center for Mind, Brain and Behavior, University of Marburg, Marburg, Germany

**Keywords:** self-awareness, anosognosia, Parkinson’s disease, mindfulness, quality of life, randomized controlled trial, intervention protocol

## Abstract

**Objective:**

This study aims to increase self-awareness in patients with Parkinson’s disease (PD) using a newly developed mindfulness-based intervention, tailored for the specific needs of PD patients. Its impact on self-awareness and patients’ daily lives is currently being evaluated.

**Background:**

Recently, the phenomenon of impaired self-awareness for motor symptoms (ISAm) and some non-motor symptoms has been described in PD. ISAm can negatively influence patients’ daily lives, e.g., by affecting therapy adherence, and is therefore the main focus of this study. The main goal is the development of IPSUM (“Insight into Parkinson’s Disease Symptoms by using Mindfulness”), a PD-specific intervention for increasing patients’ mindfulness and thereby reducing ISAm.

**Methods:**

The effectiveness of IPSUM is evaluated by comparison of an intervention group with a waitlist-control group. A pre-post design with an additional 8-week follow-up measurement is applied, resulting in three measurement points: before, directly after and 8 weeks after completing the intervention protocol. In total, up to 180 non-depressed PD patients without severe cognitive impairment (non-demented) will be included. The primary outcome is a quantitative score for measuring ISAm. Secondary outcome measures are affective changes, neuropsychological performance and self-awareness of cognition. At pre- and post-measurement an fMRI scan is performed to connect behavioral and neurobiological findings. At post- and follow-up-measurement each patient will take part in a semi-structured interview to explore IPSUM’s impact on self-awareness and patients’ everyday lives.

**Results:**

The conception of the intervention protocol is finished, the resulting 8-week program is presented in detail. It has successfully been tested in the first group of patients, their feedback so far was quite promising. Recruitment is ongoing and a first interim analysis will be performed once 30 patients have completed IPSUM.

**Conclusion:**

For the first time, the intervention protocol of IPSUM has successfully been tested in a group of PD patients. As the study goes on, more quantitative data is collected for statistical analyses to evaluate its effectiveness. More qualitative data is collected to evaluate feasibility and effectiveness. We hope for this intervention to be capable of reducing the patients’ ISAm and improving their quality of life on many levels.

## Introduction

Parkinson’s disease (PD) is one of the most common neurodegenerative disorders. Neural degeneration and loss of dopaminergic cells in the substantia nigra cause a lack of dopamine which ultimately leads to impaired motor functioning (Lill and Klein, 2017). Cardinal motor symptoms of PD include bradykinesia, rigidity and resting tremor (Postuma et al., 2015). As drug treatment commonly consists of dopamine replacement therapy, levodopa-induced dyskinesia might evolve over time (Rizek et al., 2016). Though PD is mainly classified as a movement disorder, the frequent occurrence of a wide range of non-motor symptoms has been recognized in recent years. They include sleep disturbances, autonomic dysfunction (e.g., constipation), hyposmia and psychiatric symptoms like depression, anxiety, hallucinations or impulsivity (Postuma et al., 2015).

The phenomenon of anosognosia for hemiplegia following a right hemisphere stroke is well known. The term anosognosia refers to a complete lack of self-perceived neurological or neuropsychological deficits. A partial absence of this ability is defined as impaired self-awareness (ISA) (Prigatano, 2014). Though rarely considered in the past, more recent research describes the phenomenon of impaired self-awareness for motor impairment (ISAm) in non-depressed, non-demented patients with Parkinson’s Disease (for an overview see Maier and Prigatano, 2017). ISA is associated with lower therapy adherence, as well as higher patient mortality and caregiver burden and is therefore of high clinical relevance (Prigatano, 1999; Koltai et al., 2001; Appelros et al., 2007; Turró-Garriga et al., 2013).

ISAm in PD patients has mostly been studied for levodopa-induced dyskinesia (LID) ISAm for hyperkinetic movements has also been described for other diseases such as Huntington’s disease and schizophrenia (Vitale et al., 2001; Emsley et al., 2011; Sitek et al., 2014). Depending on the used measurement method, the prevalence of ISAm for LID in PD was found in up to 91% of patients with dyskinesias (Vitale et al., 2001; Maier et al., 2015). ISAm-LID in PD is associated with higher disease duration (Amanzio et al., 2010; Maier et al., 2016), higher levodopa equivalent daily dose (Amanzio et al., 2010; Maier et al., 2016) and predominantly left-sided symptoms (Pietracupa et al., 2013). Positron emission tomography using ^18^F-Flourodeoxyglucose (FDG-PET) has shown a positive correlation between ISAm-LID and higher glucose metabolism in brain areas which are considered important for the development of LID; mainly in the left putamen, the left supplementary motor area and the left pre-supplementary motor area (Maier et al., 2016).

On the other hand, ISAm for hypokinetic movements (resting tremor and bradykinesia) is prevalent in patients with and without dopaminergic medication (ON- and OFF-state). ISAm for hypokinetic movements (ISAm-Hypo) was found in 42–54% of patients in the ON-state, and in 24–55% of patients in the OFF-state (Maier et al., 2015, 2016). Maier et al. (2016) report a positive correlation of ISAm for hypokinesias in the ON- as well as the OFF-state, but no correlation between hypokinetic movement severity and the severity of ISAm-Hypo. Concerning dopaminergic states, ISA scores in the OFF state have been associated with left-sided disease onset and worse left-sided symptoms, while in the ON-state no findings could support this relationship.

There are two theories surrounding the underlying mechanisms of ISA. It is suggested that the underlying mechanism for ISAm-Hypo might differ from the mechanism for ISAm-LID (Maier and Prigatano, 2017). For once, similar to ISA in other diseases like traumatic brain injury and Alzheimer’s Disease, right hemispheric dysfunction may cause ISAm in PD (Prigatano, 2014; Shany-Ur et al., 2014). Examining glucose metabolism in the FDG-PET study Maier et al. (2016) have found a significant association between ISA scores in the OFF-State and hypometabolism in the right inferior frontal gyrus. They also report a tendency for significance in the right insular cortex. Both regions have not only been linked to anosognosia for hemiplegia in stroke patients (Kortte et al., 2015; Moro et al., 2016), but also to ISA of overall functional competency in dementia (Shany-Ur et al., 2014). The right inferior frontal gyrus and the right insula are part of a brain network which has been associated with motor response inhibition and action monitoring and might therefore be affected in PD patients with ISAm (Fotopoulou et al., 2010; Moro et al., 2016).

Contrary to the theory of right hemispheric dysfunction, it is hypothesized that ISAm-LID emerges as a consequence of dopaminergic overstimulation (Vitale et al., 2001; Amanzio et al., 2010, 2014). While dopamine replacement therapy compensates the lack of dopamine in mesocorticolimbic pathways, and therefore enhances executive functioning, medial-prefrontal ventral-striatal circuits might be overstimulated as they are less affected by dopamine depletion (Maier and Prigatano, 2017). This might result in impaired executive functioning like attentional set shifting, response inhibition and performance monitoring. Amanzio et al. (2011) suggested that a dysfunction of the cingulate cortex, which is typically involved in action and performance monitoring, contributes to the phenomenon of ISA. Although ISAm-LID has been associated with lower cognitive performance by some researchers, contradictory findings have not shown any correlation of ISAm-LID and cognitive or executive performance in neuropsychological tests (Maier et al., 2012, 2016; Pietracupa et al., 2013).

Despite the growing research interest in ISA for motor symptoms in PD, it is worth mentioning that ISA might also exist in regards of cognitive impairment (ISAc), mainly for memory and executive impairment (Kudlicka et al., 2013; Lehrner et al., 2015). Pillai et al. (2018) conclude that impaired self-appraisal for cognitive functioning is equally likely to occur in PD patients with mild cognitive impairment (MCI) and patients with amnestic MCI, which often progresses to Alzheimer’s Disease. Orfei et al. (2018) have highlighted the importance of the general level of cognitive functioning. They found higher anosognosia in PD patients with dementia and multi-domain MCI compared to PD patients with single-domain MCI or normal cognitive functioning. Interestingly, greater anosognosia might also be associated with depression and lower executive functioning. It has to be noted though, that other researchers have not found any evidence for ISAc in PD (Starkstein et al., 1996; Sitek et al., 2011, 2013; Koerts et al., 2012). In their review, Maier and Prigatano (2017) have suspected the use of different methods to assess ISAc, sample differences, as well as the potential importance of a present cognitive impairment to be reasons for these conflicting results. Future research should therefore comprise extensive neuropsychological test batteries and imaging data to get further insight into the phenomenon of ISAc. It is worth mentioning that newer research has already taken some of these aspects into consideration (Orfei et al., 2018; Pillai et al., 2018).

To study anosognosia for memory impairment in Alzheimer’s Disease Vannini et al. (2017) calculated an anosognosia index to quantify the discrepancy between subjective and objective memory scores. Using this method, they found decreased memory awareness in patients with amnestic mild cognitive impairment in comparison to a healthy control group. Among others, they also reported an association between lower memory awareness and reduced glucose metabolism in the posterior cingulate cortices and the hippocampus. Maier et al. (in preparation) applied a similar method to study differences of cognitive awareness between PD patients with and without MCI and healthy controls. For PD patients as a whole, they found an association between higher impairment of awareness and reduced metabolism in FDG-PET in the anterior and mid-Cingular cortices. Specifically, for PD patients with MCI they report the same association in the mid-Cingular cortex as well as the right superior temporal area and parts of the adjacent insular cortex. Taking neurobiological findings of ISAm and ISAc in consideration, the cingulate gyrus, as well as the (right) insula seem to play an important role regarding the general development of impaired self-awareness.

The concept of mindfulness originated in Buddhism, but has grown in popularity in western civilization and scientific research for several years (Kabat-Zinn, 2013; Fox et al., 2016). It has been formerly described as “paying attention in a particular way: on purpose, in the present moment, and non-judgementally” (Kabat-Zinn, 2004, p. 4). Mindfulness can be understood as a kind of personality trait, which can be improved by regular formal or informal mindfulness practice (Keng et al., 2011). While formal practice involves meditation or yoga practice, informal practice comprises all sort of daily activity which is performed while maintaining the described mindful attitude (Kabat-Zinn, 2013; Schug, 2016).

In general, mindfulness is strongly associated with variables of psychological health like quality of life, feelings of vitality and autonomy or optimism (Brown and Ryan, 2003; Rasmussen and Pidgeon, 2011). On the other hand, it is negatively associated with depression, social anxiety or other psychiatric symptoms (Brown and Ryan, 2003; Baer et al., 2006; Dekeyser et al., 2008; Cash and Whittingham, 2010; Rasmussen and Pidgeon, 2011). For more detailed information see Keng et al. (2011). Similar results have been reported for meditation practice alone, which is an essential part of most intervention concepts. While meditation practice seems to reduce negative emotions, like anxiety and stress, it might also enhance cognitive performance of attention and self-reflection (Sedlmeier et al., 2012). Further studies also hint at possible positive implications of mindfulness training on attention and possibly working memory and executive functioning (Chiesa et al., 2011).

Other studies also suggest positive implications in regards of body awareness. For example, early qualitative studies indicate a more positive self-representation and acceptance toward oneself as well as higher responsivity and intensity of body and emotional perception caused by regular yoga practice (Emavardhana and Tori, 1997; Daubenmier, 2005; Impett et al., 2006; Dittmann and Freedman, 2009). Additionally, long-term meditators showed an improved awareness and interpretation of body states (Tang et al., 2015), a higher coherence of emotional perception and physiological arousal, as well as more sensitivity for body sensations (Sze et al., 2010). In a study conducted by Fox et al. (2012), meditators reported more intense body sensations after completing a guided body scan meditation (in which the meditator focuses his/her attention systematically on different parts of the body) compared to a control group.

These behavioral findings regarding self-awareness and perception are also reflected on a neurobiological level. Early findings have suggested that meditators, compared to matched controls, display higher cortical thickness in the right prefrontal cortex and right insula. These areas have been associated with attention, introspection and processing of sensory stimuli (Lazar et al., 2005). In addition to higher cortical thickness, in a meta-analysis considering 21 studies, Fox et al. (2014) identified differences in volume and density of gray and white matter in various regions. Regions linked to self-awareness include the bilateral insula, somatomotoric cortices, the rostrolateral prefrontal cortex, and the anterior and mid-cingulate cortices. These regions have been mostly associated with abilities of introspection, metacognitive and body-oriented perception and self-regulation. According to the authors, especially changes in the area of the insula are central to general meditation practice, independently of the type of meditation practiced (Fox et al., 2014, p. 61). Although most studies have focused on cross-sectional comparisons between long-term meditators and novices, it is assumed that the effects regarding areas like the prefrontal cortex, the cingulate gyrus and the insula might be just as significant after only 8 weeks of mindfulness training (Gotink et al., 2016).

In a clinical context, many psychological mindfulness-based interventions exist. The most popular and most cited program is called Mindfulness-Based Stress Reduction (MBSR), which was developed by Jon Kabat-Zinn to improve stress and pain management in patients with chronic pain (Kabat-Zinn, 1982). In fact, the effects of MBSR and other mindfulness-based interventions have been studied in various physical chronic diseases, like Multiple Sclerosis, chronic pain or cancer. For those patient groups, positive effects have been found especially in regards of quality of life, stress and fatigue (Ott et al., 2006; Grossman et al., 2010; Rosenzweig et al., 2010). Larouche et al. (2015) have suggested that mindfulness-based interventions might slow down cognitive decline in patients with Alzheimer’s Disease.

In PD, some studies have already investigated the effects of mindfulness interventions in pre-post-designs. Participating in an intervention has led to an increase of mindfulness *per se*, an improvement of quality of life, as well as a reduction of negative emotions like depression, anxiety and stress (Pickut et al., 2015; Advocat et al., 2016; Cash et al., 2016; Dissanayaka et al., 2016). Cognitive functioning might also be positively influenced as improvements of attention, mental flexibility and self-reported ability of speaking have been reported (Cash et al., 2016). The only neurobiological study in a PD population investigating mindfulness was published by B. A. Pickut et al. (2013) who found an increase of gray matter density in the right and left hippocampus and part of the right amygdala. However, the authors postulate the need for more studies to clarify the neurobiological changes caused by mindfulness training in PD. Moreover, McLean et al. (2017) criticized methodological flaws for most behavioral studies, too. Hence, they have not been able to conduct a meta-analysis regarding the impact of mindfulness in PD patients and also emphasize the need for more studies of high quality. In addition, Rodgers et al. (2019) recently conducted a pilot trial to test the efficacy of a modified protocol of mindfulness-based cognitive therapy (MBCT) for reducing depression. For patients participating in a 6-week MBCT intervention, they report a significant reduction in depressive symptoms. Though no reduction of anxiety or improvement of quality of life was found, the results of this study suggest that mindfulness-based interventions can potentially be helpful to fight depression in PD patients.

To the best of our knowledge, so far, no study has investigated the effects of mindfulness on ISA in PD patients. The aim of this study is to get better insight into the effects of mindfulness training on impaired self-awareness in PD. Therefore, we present IPSUM, a protocol for a newly developed mindfulness-based intervention to increase self-awareness in PD. IPSUM, an acronym for “Insight into Parkinson’s Disease Symptoms by using Mindfulness,” is an 8-week intervention, whose development has been influenced by existing mindfulness-based programs as well as previously reported experiences with mindfulness interventions in groups of PD patients. IPSUM is innovative because (1) it is tailored for the specific needs of PD patients (2) educates about the use and practicability of mindfulness specifically in PD and (3) has a larger focus on the aspect of self-awareness and its implications for daily living compared to other mindfulness interventions. It takes into account the specific needs and impairments of PD patients, such as a reduced attention span, impaired executive functioning and lesser mobility.

We expect this new intervention to increase mainly impaired self-awareness for motor symptoms, but also to affect cognitive ISA. Based on the described general mindfulness literature, we assume several factors involved for mindfulness training to be an effective way to increase self-awareness in PD patients. For once, training the ability to describe one’s own perception (instead of judging it) might affect the patients’ view on their own body (Daubenmier, 2005). As they start being more focused on the present moment (e.g., during mindfulness meditation and yoga exercises), they also should be more aware of their body movements and posture as well as other internal sensations (including their thoughts and emotions). Achieving and maintaining this perceptional shift might be facilitated by increasing acceptance toward themselves and their disability, regular attention training in form of mindfulness meditation and improved emotion regulation of unpleasant emotions (Cayoun, 2005; Tang et al., 2015). As described earlier, neurobiological studies reported structural changes of brain regions associated with abilities of introspection, meta-cognitive and body-oriented perception and processing of sensory stimuli (Lazar et al., 2005; Fox et al., 2014). Therefore, we expect neurobiological changes caused by regular mindfulness training to strongly support the training of self-awareness. As the cingulate gyrus and the insula have shown to be central to ISAm and seem to be influenced by mindfulness practice, we are particularly interested in those areas.

In addition, as it seems typical for mindfulness interventions, we hope to find an increase of several aspects of emotional well-being (e.g., quality of life) while reducing negative emotions like depression, anxiety or stress and other PD-related non-motor symptoms (e.g., apathy or impulsivity). As mindfulness-related research has indicated, an increase of cognitive performance in dimensions that are impaired in PD, mainly attention and executive functioning, might also be possible. As neurobiological research for this specific field is practically non-existent, we also plan to perform resting-state fMRI and structural MRI scans to link behavioral and neurobiological data. To get more detailed information about changes relevant for the patients’ everyday life, a short semi-structured interview is planned.

## Methods

Patients with idiopathic PD (diagnosed according to the Movement Disorder Society PD criteria (Postuma et al., 2015) are recruited from the Department of Neurology, University Hospital Marburg, Germany. Up to 180 patients between 45 and 85 years of age will be included.

Exclusion criteria are depression [Beck Depression Inventory-2 – BDI-II score >19 (Beck et al., 1996; Hautzinger et al., 2006)], dementia [Parkinson Neuropsychometric Dementia Assessment – PANDA score <15 (Kalbe et al., 2008)] and a clinical diagnosis of additional severe neurological or psychiatric disorders. Patients with an advanced disease stage [i.e., Hoehn and Yahr scale, stage 5 (Hoehn and Yahr, 1967)] are excluded as they are not expected to be able to perform practical exercises during the intervention. Additionally, patients with prior regular experience in meditation or yoga are excluded as this study seeks to examine mindfulness novices. As this study seeks to improve an impaired self-awareness, patients must show signs of ISAm. This is checked by using a short screening tool. For all patients, antiparkinsonian medication is registered and has to be unchanged for at least 2 weeks prior to baseline measurement. Furthermore, patients’ eligibility to undergo an MRI scan is checked (though this is no exclusion criterion). The study has been approved by the local ethics committee of the University Hospital Marburg (Study number: 119/18) and registered at the German Clinical Trials Register (DRKS00015807). All patients have to give written informed consent prior to participation.

### Study Design

**FIGURE 1:**
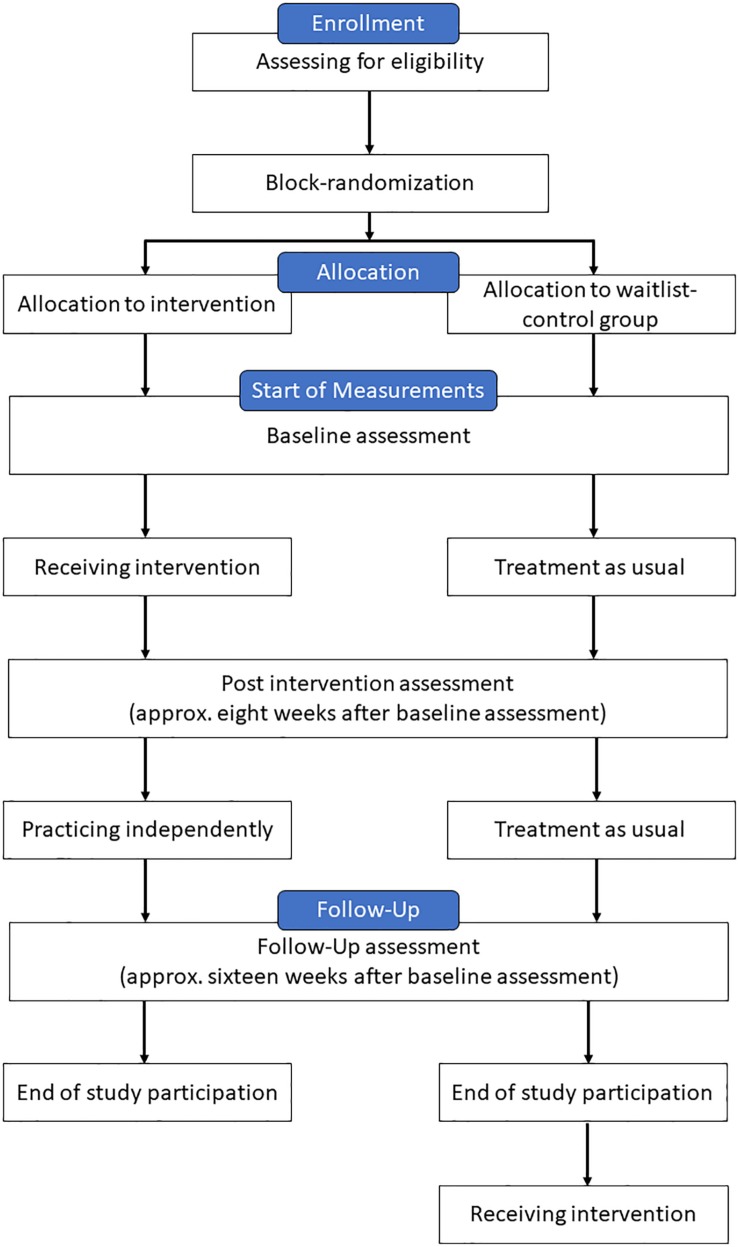
Course of the study for each patient.

To evaluate the effectiveness of IPSUM on ISAm an adaptive pre-post-design is applied. First, eligible patients are randomized into an intervention group and a waitlist control group. After approximately eight to twelve patients have been successfully recruited, they are randomized by a computer program. Patients of the intervention group are measured at three points in time: before, directly after and 8 weeks after the intervention has ended. As stated before, the intervention takes place between the first two measurements and continues for 8 weeks. Patients of the control group are measured at the same time intervals, but do not take part in the intervention and are therefore treated as usual. For ethical reasons those patients can participate in the intervention after the study has ended. Figure 1 gives an overview of the course of the study.

**FIGURE 2:**
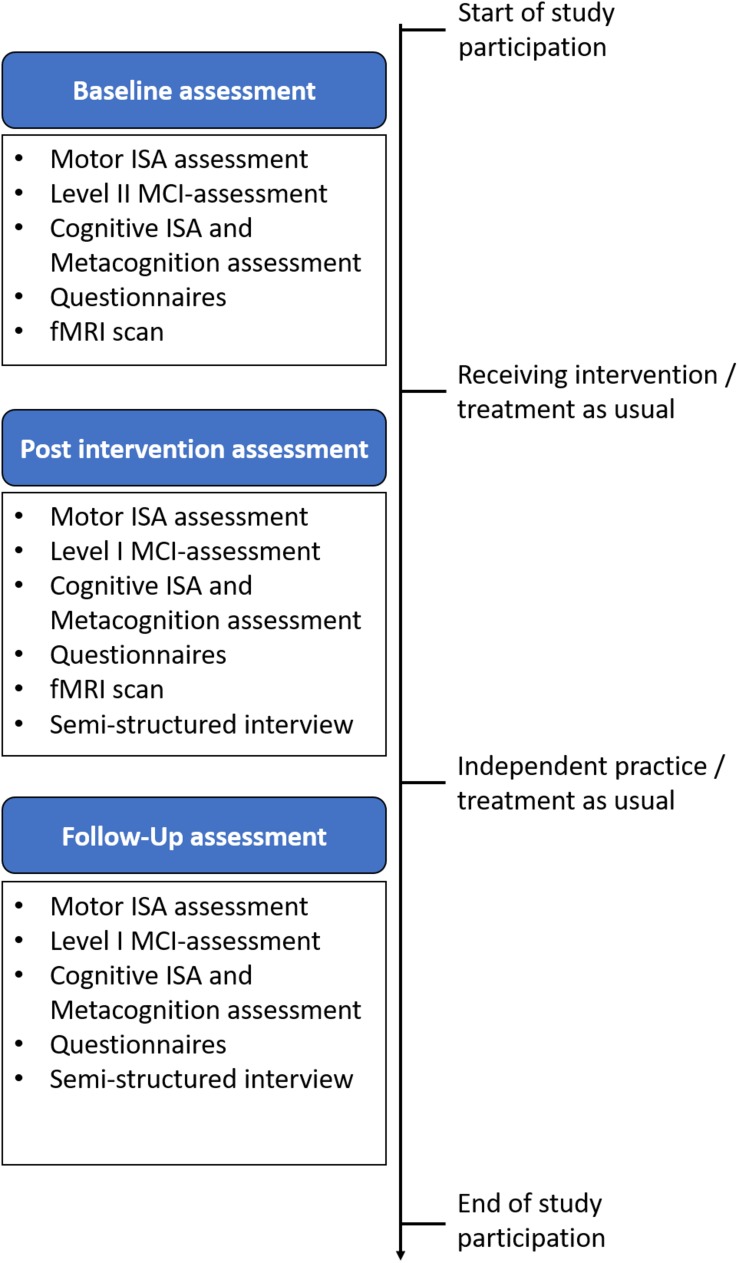
Comprehensive overview of all three measurement points.

At all three points in time ISAm is evaluated. Additionally, patients complete several questionnaires for emotional well-being. Cognitive functions are assessed using an elaborate neuropsychological test battery. Up to 30 patients will undergo a resting-state fMRI and structural MRI scan. Patients who have completed the intervention protocol can take part in an interview to report possible changes in their everyday life. For clarification, Figure 2 gives an overview of the tests performed at each measurement point. All patients, independent of their study group, complete all tests in the same order. For some neuropsychological tests parallel forms are available. For those tests, the applied version at each time point is randomly selected after a patient’s group assignment. It has to be noted, that all measurements are performed during the medication ON-state. Hereafter, primary and secondary outcomes, as well as all measurement procedures are further elaborated.

### Outcome Measures

The main goal of this study is to increase self-awareness for motor symptoms. Therefore, the primary outcome for efficacy testing is a global ISAm-score. To be precise, mainly its change from baseline to post-intervention assessment is analyzed to evaluate the effectiveness of IPSUM. Changes of ISAm from baseline to follow-up are considered as a secondary outcome. However, we determined several other secondary outcomes like general neuropsychological test performance, the congruence between neuropsychological test performance and subjective daily life impairment (ISAc) and the congruence between objective neuropsychological test performance and subjective test performance (metacognition). Also, changes of several PD related symptoms (depression, apathy, impulsivity, sleeping problems) and affective states (stress, anxiety, quality of life, mindfulness) are analyzed. The aforementioned secondary outcomes comprise all changes from baseline to post measurement as well as changes from baseline to follow-up measurement. Additionally, structural neurobiological changes in the Insula and Cingulate Cortices, as well as any kind of reported subjective changes during the semi-structured interview are also considered when IPSUM’s effectiveness is evaluated.

### Motor ISA-Assessment

A common method to evaluate self-awareness is by comparing self and outside assessments. In this study we use the recently developed and validated measurement method by Maier et al. (2015). The patient is shown several video clips where a healthy person demonstrates various movements. The task for the patient is to repeat the movement him/herself as seen in the video clip. The following 15 symptoms are examined:

1. Sitting on a chair: resting tremor (left and right hand), dyskinesia.
2. Right hand pronation-supination: speed and amplitude of movement, dyskinesia.
3. Left hand pronation-supination: speed and amplitude of movement, dyskinesia.
4. Arising from a chair: resting tremor (left and right hand), dyskinesia.
5. Walking down an aisle: resting tremor (left and right hand), dyskinesia.

The symptoms are distributed across the four subscales dyskinesia, resting tremor right hand, resting tremor left hand and bradykinesia. Following each task, the patient rates his/her performance on a dichotomous scale whether the movement was impaired or not. Regarding the perception of a symptom, in total each patient answers 15 questions with either yes or no. The whole procedure is recorded by video. Later, independent raters evaluate the movements as well. In case of any discrepancies – if the patient does not see any impairment, although the raters do – an impaired self-awareness is noted. For each symptom, irrespective of the patient’s awareness, they also rate its severity according to UPDRS-III (values range 0–4; 0 = normal/absent; 1 = mild; 2 = moderate; 3 = severe; 4 = unable to perform). This procedure allows the calculation of two independent scores: one score for overall motor impairment severity and a second score for ISAm severity. While the motor impairment score is calculated by summing up all motor severity ratings, the ISAm score is calculated by summing up all motor severity ratings of the potential 15 discrepancies. Hence, both scores can vary between 0–60. In the medication ON-state the ISAm global score can also be divided into subscores for impaired self-awareness for hypokinetic symptoms (ISAm-Hypo for tremor and bradykinesia) and hyperkinetic symptoms (ISAm-LID).

### Assessment of Cognitive Performance, Cognitive-ISA and Metacognition

Each patient’s cognitive performance is evaluated at baseline measurement. To identify patients with mild cognitive impairment (MCI), MCI-Level II-Assessment is applied. To the best of our knowledge, no established neuropsychological test battery exists to perform level II testing. Therefore, we have compiled a test battery ourselves, while also taking into account the guidelines of diagnostic criteria for MCI in PD (Litvan et al., 2012). To differentiate between single- and multiple domains of PD-MCI, for research purposes it has been strongly recommended to use two different neuropsychological tests for each cognitive domain. Table 1 specifies the neuropsychological tests used for this purpose.

**TABLE 1:** Overview of the applied neuropsychological test battery.

Domain	Test I	Test II
Attention/Working memory	TAP: Sustained attention	WMS-R: Digit span forward/backwards
Executive Functioning	RWT: Alternating semantic verbal fluency	Trail Making Test A + B
Language	WAIS-IV: Similarities	WAIS-IV: Vocabulary
Memory	VLMT	ECFT-MI: Recognition Trial
Visuospatial Ability	WMS-R: Spatial Span forward/backward	ECFT-MI: Matching Trial

*ECFT-MI: Extended Complex Figure Test – Motor Independent Version (Fastenau, 1996); RWT: Regensburg verbal fluency test (Aschenbrenner et al., 2001); TAP: test battery for attention (Zimmermann and Fimm, 2002); VLMT: Verbal learning and memory test (Helmstadter et al., 2001); WAIS-IV: Wechsler Adult Intelligence Scale – Fourth Edition (Wechsler, 2008); WMS-R: Wechsler Memory Scale-Revised (Härting et al., 2000).*

For time-economic reasons and to minimize patient strain, full MCI-Level II-Assessment cannot be performed during post- and follow-up measurement. To investigate the effects of mindfulness on cognitive performance regardless, one test for each domain is applied at these time points. For this purpose, tests listed in the column “Test I” are used. Each patient also completes the Montreal Cognitive Assessment [MoCA (Nasreddine et al., 2005)]. As mentioned before, some neuropsychological tests offer parallel versions. To minimize training effects caused by performing the same test more than once, the applied test version of the Regensburg verbal fluency test, the Verbal learning and memory test and the MoCA is chosen randomly for each patient. It is possible that the same test version is assigned to a patient more than once. In addition, neuropsychological data is used to evaluate two kinds of cognitive awareness. For one, self-awareness for performance level in daily life and secondly, metacognition for cognitive performance in neuropsychological tests.

#### Impaired Self-Awareness of Cognition

To investigate impaired self-awareness of cognition (ISAc), subjective cognitive impairment in everyday life is compared with the more objective performance in neuropsychological tests. Subjective cognitive impairment in general is assessed by using the Cognitive Failures Questionnaire [CFQ (Broadbent et al., 1982)]. Additionally, for impairment of executive functions the Dysexecutive Questionnaire [DEX (Wilson et al., 1998)] is applied. Higher scores reflect higher subjective impairment in both questionnaires. For further statistical analysis, questionnaire and neuropsychological test raw data is transformed into standardized z-scores to allow better comparison. A delta score is calculated by subtracting subjective scores from objective scores. Beforehand, depending on the analytical objective, the transformed values of all neuropsychological tests are either summed up for reflecting general cognitive performance, or only verbal fluency performance is taken into account to reflect executive cognitive performance. Impaired self-awareness of cognition occurs if objective neuropsychological test performance appears to be worse than subjective impairment in everyday life. This is reflected by a positive delta score. A similar method has been used before to study anosognosia of memory deficits in Alzheimer’s Disease (Vannini et al., 2017).

#### Metacognition

In addition to comparing subjective cognitive performance in daily life, we also plan to compare the patients’ objective cognitive performance (according to normative value) to their estimated performance in neuropsychological tests. Therefore, each patient is asked to rate his/her performance compared to a healthy person of the same age, right after completing a neuropsychological test. On a 5-point Likert scale, the patient rates whether his/her performance is equal to the performance of the upper 20, 40, 60 or lower 20 or 40 percent of people the same age. By doing so, the patients are required to observe themselves from a metacognitive perspective. If the patients rate their own performance better than their objective test performance, the ability of metacognitive observation might be impaired.

### Questionnaires

To evaluate the effects of IPSUM on emotional well-being as well as other non-motor symptoms of PD, all patients are asked to complete several questionnaires. All questionnaires are filled out at all three measurement points. PD related symptoms of interest are depression [BDI-2], Apathy [Apathy Evaluation Scale – AES (Marin et al., 1991)], Impulsivity [Questionnaire for Impulsive-Compulsive Disorders in PD – QUIP (Probst et al., 2014)] and Sleeping Problems [Parkinson’s Diseases Sleep Scale-2 – PDSS-2 (Trenkwalder et al., 2011)]. Furthermore, to study changes of emotional well-being, experienced stress [Perceived Stress Questionnaire-20 – PSQ-20 (Fliege et al., 2005)], state and trait anxiety [State-Trait Anxiety Inventory – STAI (Spielberger, 2010)] and quality of life [Parkinson’s Disease Quality of Life – PDQ-39 (Berger et al., 1999)] are assessed. Data about subjective cognitive impairment in general [CFQ] and specifically for executive function [DEX] is needed to evaluate ISAc, as described above. Since this study intends to evaluate the effects of a mindfulness based intervention, trait mindfulness [German Version of the Five Facet Mindfulness Questionnaire – FFMQ-D (Marin et al., 1991; Michalak et al., 2016)] is also assessed.

### Imaging

Eligible patients are asked to undergo an optional MRI scan during baseline and post measurement to study potential neurobiological changes induced by the intervention. Additionally, the neurobiology of ISAm and ISAc can further be studied. The MRI protocol comprises a T1-weighted structural scan for morphometric analysis, a blood oxygen level-dependent (BOLD) resting state time series to analyze functional connectivity, as well as a diffusion-weighted sequence for tractography. Scans will be acquired on a 3T Siemens Trio MRI scanner. Currently up to 30 patients (15 per group) are planned to participate in the imaging measurement of this study.

### Semi-Structured Interview

Up to this date, the applied quantitative measurement of ISAm has not been used for longitudinal studies. Therefore, it is uncertain if the instrument is sensitive to changes over time. Because of that, every patient who successfully completed the intervention protocol can take part in a semi-structured interview focusing on the possible changes they noticed over the last weeks (Supplementary Table S1). This also includes possible side effects caused by the intervention. The interview takes place during post- and follow-up measurement. By collecting qualitative data, we hope to get a more detailed insight into changes induced by mindfulness, especially in regards of self-awareness, and their relevance for the patient’s everyday life. Additionally, feedback regarding the intervention itself can be collected to further improve the training protocol in the future.

### Intervention Protocol

IPSUM is an 8 weeks long, group-based intervention which seeks to improve mindfulness and self-awareness of motor symptoms in patients with PD. In this study, all training sessions are held by a psychologist (main author T.B.). The concept is designed for groups of 4–8 patients and consists of 8 weekly sessions of approximately 2 h of duration. The main topic of a session differs from week to week. Table 2 gives an overview of all weekly topics.

**TABLE 2:** Session Overview.

Week	Topic	Main goal
1	Introduction to the concept of mindfulness	Understanding the general concept in theory and by practice
2	The power of breathing	Understanding that we can only control our behavior (e.g., by experiencing the importance of breathing)
3	Thoughts and appraisals	Introduction to the concept of defusion from one’s own thoughts and appraisals and the importance of observing them
4	How to deal with emotions	Learning about the purpose of pleasant and unpleasant emotions and ways of self-care
5	Stabilization of mindful practice	Repetition of previously gained knowledge as well as solving current problems with daily practice
6	Mindfulness and stress resilience	Learning about the use of mindfulness techniques in regards of stress reduction
7	Moving meditation	Performing the complete sequence of sitting yoga composed of movements learned during the training
8	Closing session: A new beginning	Evaluation of the whole training participation and to find solutions for persisting problems of mindful practice to facilitate independent practice after the intervention has ended

While each session’s general topic is different, various recurring elements can be highlighted. For once, each week contains of a short theoretical input which fits the weekly topic. To account for possible impairment of attention, concentration or general cognition, this aspect will not exceed a duration of 15 min. A practical exercise which builds on the theoretical input is performed.

Secondly, patients practice guided mindfulness meditation in a group setting. To prevent patients from falling asleep or cancel meditation practice, various elements of gentle movement and muscle tensing and releasing are included. Additionally, some basic yoga movements are taught. To account for the impaired mobility of patients with PD, sitting yoga on a chair is practiced. At the end of the intervention each patient will have learned a simple yoga sequence which they can also perform at home. As the ability to describe one’s own experience (instead of judging it) is central to the concept of mindfulness, this part is extended by a small sensory exercise. Here, patients are handed a different object each week (e.g., wool or a heat pad) and are asked to describe it as detailed as possible using all five senses. As noted in a previous study of a mindfulness intervention with PD patients, patients find it helpful to focus on external stimuli instead of internal sensations alone (Birtwell et al., 2017).

Previous research also has shown that the time invested for mindfulness practice is of high importance. Therefore, each week patients are asked to practice body scan and mindfulness meditation at home using an audio CD with guided instructions. Since all participants are meditation beginners and also might have attentional deficits, practice time is raised each week, up to 30 min a day. To support the implementation of mindfulness into the patients’ everyday life, they are also asked to perform an informal mindfulness exercise which changes weekly, e.g., mindful walking or mindful eating. If possible, the time used for mindful practice should be noted in a journal for future analyses.

Starting in week two, each session will begin with a reflection of the week before. Here, the patients get the opportunity to talk about their experiences with the previous week’s homework and possible difficulties they might have encountered. The group setting is expected to be quite helpful for sharing common experience among the patients and getting support to overcome possible problems that may arise during daily practice. At the end of each session, each patient receives a brief written summary containing all relevant information of the theoretical input to allow reviewing specific details of the newly learned information as needed and to support independent practice at home. To continue mindfulness training at home, patients are asked to further practice 30 min of daily mindfulness meditation in addition to improving their informal mindful practice. To ensure compliance, patients are asked to note down all performed mindfulness exercises in tables contained in their written summary for another 8 weeks.

### Planned Statistical Analyses

The results of a power analysis suggest, that a total of up to 166 patients (83 patients per group) might be needed to detect a significant effect. By applying a group sequential study design the number of patients needed is raised to 180 to allow for interim analyses to be performed after including 60 and 120 patients, respectively. To the best of our knowledge the motor ISA-Assessment has not been applied in a longitudinal study design. Depending on the results of these interim analyses or due to economic reasons, the study might be stopped beforehand.

Demographic and baseline characteristics will be compared using *t* tests or other non-parametric methods, if necessary. To analyze most of the primary and secondary outcomes, performing repeated-measures mixed model analyses of covariance will be essential. Independent variables will be group (intervention and waitlist) and time (pre, post and 8-week follow-up) and also their interaction. Baseline measure, the amount of medication (levodopa equivalent daily dose) and depression scores will be included as covariates. To specifically analyze ISAm scores, which are the main outcome, and to determine their degree of objectiveness, inter-rater reliability will be computed for each measurement point in time.

In order to get more insight into possible mechanisms of mindfulness, ISA and their connection to each other, several moderation and mediation analyses are planned. For example, we expect the number of completed training sessions, motor symptom severity and patients’ general cognitive performance level to be possible moderating factors affecting mindfulness scores itself and/or the relationship between mindfulness and ISA. Among others, mediating factors considered will be cognitive performance of sustained attention and questionnaire scores of negative emotions like anxiety and depression. Of course, neurobiological changes will be included as well.

When the effectiveness of a treatment is evaluated, the question of how to deal with dropout patients has to be considered. If a patient cannot conclude his or her study participation, the reason and time of drop out will be documented. To still be able to include as many patients as possible in the analyses, the intention-to-treat analysis is preferred. For missing values, missing at random-analyses will be performed. In case of positive results, maximum likelihood methods will be applied. Data will be analyzed using SPSS, version 26.0 (IBM Corp., Armonk, NY, United States). To analyze qualitative data collected during semi-structured interviews, qualitative content analysis will be used (Mayring, 2015).

Imaging fMRI data will be analyzed using SPM12 (Penny et al., 2007). Functional connectivity of the left and right anterior insula and the anterior cingulate – central nodes of the salience network which have repeatedly been described to be altered following mindfulness interventions – will be analyzed by comparing pre to post measurements within and between groups, focusing on connectivity with the medial prefrontal cortex and the default mode network (Kilpatrick et al., 2011; Doll et al., 2015). Voxel-based morphometry will be applied to perform pre-post between-group comparisons of gray matter density in the same regions. Diffusion MRI will be used to analyze training effects on fractional anisotropy of the anterior-superior parts of the corona radiata and corpus callosum, following results reported after a similar intervention in healthy subjects (Tang et al., 2010). Neural correlates of impaired self-awareness (motor and cognitive) will be assessed based on previous findings in FDG-PET, with the cingulate gyrus and right insular cortex as regions of interest in morphometric and functional connectivity analyses.

## Discussion

The phenomenon of anosognosia has been described for various neurological diseases. Recently a less extreme version, the phenomenon of impaired self-awareness, has been observed in non-demented, non-depressed patients with PD. While it has been mostly reported for motor symptoms, it might also occur for cognitive or other non-motor symptoms. Based on scientific research, mindfulness training is suggested as a possible therapy concept. However, existing mindfulness interventions often do not consider the specific needs of patients with PD, e.g., decreased mobility or attention deficits. Therefore, we developed IPSUM, a new mindfulness-based intervention. IPSUM does not only consider the special needs of PD patients, but also has a larger focus on the aspect of self-awareness compared to other mindfulness interventions. While its feasibility and effectiveness in regards to impaired self-awareness of motor symptoms are the primary objectives of this study, we also want to evaluate changes of cognitive performance and self-awareness, as well as other non-motor symptoms and emotional well-being. As some patients undergo an fMRI scan, we hope to get better insight into the neurobiological changes caused by mindfulness training in PD patients with impaired self-awareness. Additionally, to evaluate the feasibility of IPSUM and to investigate the impact of mindfulness on the patient’s daily life, a semi-structured interview is conducted.

### The Need for a Tailored Mindfulness Intervention Protocol

As stated before the concept of MBSR has been widely applied and studied in a variety of patient groups. Indeed, previous studies, which focused on the effects of mindfulness in PD, have administered the MBSR program or a variation of it. We intended to create a new intervention protocol to meet the specific needs of PD patients, which are mostly elderly people, many of whom have never heard of the concept of mindfulness, and usually do not have any prior experience with yoga, meditation and/or mindfulness exercises. Considering those needs has led to another mindfulness concept which mainly differs from the MBSR concept in three ways.

For once, the main goal is different. While MBSR mainly deals with pain and stress, IPSUM seeks to improve self-awareness of motor symptoms which are very specific for and almost exclusively occur in PD. Using the IPSUM protocol, it is expected to achieve some sort of stress reduction, too, as the practiced exercises are partly overlapping (e.g., mindful meditation, body scan). However, the instructions in IPSUM often remind the patient to focus on specific motor symptoms and their observation, if a symptom is present indeed. The repetitive focus on motor symptoms is expected to increase the patients’ awareness for their symptoms and thus fulfill the main goal.

Secondly, to consider possible problems of executive functioning (e.g., problems with planning) a general structure is persistent throughout the entire intervention to provide some sort of orientation for the patient. For example, for each day another suggestion for informal mindful practice is given. Additionally, the patient is not explicitly asked to practice without guided instructions (which they would be asked to do in MBSR). However, he or she still has the option to do so, if he or she wants to.

Thirdly, general session duration is shorter and all parts of a session usually do not last longer than 15 min because of possible attention problems. During the weekly session small movement breaks are implemented to increase the patients’ vigilance. Homework practice duration is gradually increased to allow for an early sense of accomplishment despite possible cognitive impairment. The duration interval then is gradually increased to up to 30 min of practice per day. In contrast to the MBSR concept, the IPSUM protocol does not include a 6 h “day of mindfulness” at all as this might be too demanding.

Given these reasons, we felt the need to conceptualize a tailored mindfulness protocol for patients with PD.

### Strengths of This Study

This study is conducted with the expectation of further insight into the phenomenon of impaired self-awareness in PD and its relation to practiced mindfulness. Therefore, in the following the study’s strengths are highlighted.

For once, this is the first project to study the relationship between self-awareness and mindfulness in PD which also collects longitudinal quantitative and qualitative behavioral and neurobiological data. Though the planned sample size of up to 180 patients is quite large, it has to be noted that the study might be stopped beforehand due to economic reasons or a lack of eligible patients. However, sample sizes of other studies were much smaller (usually around 10 to 15 patients with one study including around 60 patients). Despite a small sample size, they also found significant effects for mindfulness related aspects (Advocat et al., 2013; Pickut et al., 2013; Cash et al., 2016; Dissanayaka et al., 2016; Birtwell et al., 2017). Additionally, similar to other studies the interventional effect is compared to a waitlist-control group but not an active control group. The planned training duration of 8 weeks are standard and, based on described literature, should be adequate to achieve significant changes. If a significant impact is found in this sample, an active control group and longer time intervals between measurements should be considered for further evaluation of the training protocol.

A main part of this study is the evaluation of ISAm, which is done by using a psychometric evaluated tool developed by Maier et al. (2015). This method is similar to the evaluation of ISAm in previous studies (Maier et al., 2016). In our study, however, it is for the first time assessed with repeated measurements. Also, we only assess ISAm during the medication ON-state, but not in the OFF-state. Not taking their medication would be another heavy strain for each patient, and we therefore decided to refrain from OFF-state evaluation of ISAm. Additionally, the ON-state is more accurate in reflecting the patient’s daily life situation. As we also focus on the implications of mindfulness in everyday life during an interview, we get the opportunity to combine qualitative and quantitative data. Future studies might also focus on the evaluation of OFF-state ISAm. As this is also the first time this method is used in a longitudinal study, its sensitivity to change is still unknown.

The measurement of cognitive performance is quite comprehensive as a full MCI-Level II-Assessment is applied at baseline. Though this test battery is self-compiled, it does meet established criteria for the diagnosis of MCI. However, due to time-economic reasons and patient strain the full test battery cannot be applied at post- and follow-up-measurement. Instead one neuropsychological test for each domain is assessed to make sure changes of cognition can be investigated. Regarding the evaluation of ISAc, it has to be noted that the described analyses are not validated, but have been used before (Vannini et al., 2017).

## Conclusion

In conclusion, we expect this study to prove the feasibility and preliminary effectiveness of IPSUM, a newly developed mindfulness-based group intervention for the specific needs of PD patients, by increasing self-awareness of motor and cognitive symptoms and also increasing the patient’s quality of life on many levels.

## Ethics Statement

The studies involving human participants were reviewed and approved by Local Ethics Committee of the University Hospital Marburg. The patients/participants provided their written informed consent to participate in this study.

## Author Contributions

TB, CE, and FM contributed to the conception and design of the study. TB developed the intervention protocol with support of AG. TB wrote the first draft of the manuscript. All authors contributed to manuscript revision, read and approved the submitted version.

## Conflict of Interest

In the last 12 months CE has received speaker’s or consulting honoraria from AbbVie Inc., Bial Inc., and Daiichi Sankyo Inc. The remaining authors declare that the research was conducted in the absence of any commercial or financial relationships that could be construed as a potential conflict of interest.
