# Supplementary material for: Improving Self-Awareness of Motor Symptoms in Patients With Parkinson’s Disease by Using Mindfulness – A Study Protocol for a Randomized Controlled Trial
Source: Front Psychol. 2020 Apr 17;11:743. doi: 10.3389/fpsyg.2020.00743 (PMC7180229; doi:10.3389/fpsyg.2020.00743)
Supplement: Supplementary file 1 [file Table_1.DOCX]

**Interview guide for semi-structured interviews**

| **Key Question** | **Content Aspect** | **Additional Questions** |
| --- | --- | --- |
| **Introduction: Emotional well-being**  Imagine, every person had an internal speedometer that shows how stressed or relaxed we are (1: very relaxed; 10: very stressed). What would your speedometer show right now? | - Current level of relaxation - General level of well-being - Specific examples for positive/negative moments - Coping strategy for negative moments | Why would you think so?  Can your current status be generalized for your everyday life?  Looking back on last week, was there a situation where you felt (very) good or bad? Why?  What did you do in this situation? |
| **Self-care**  Looking back on last week, did you do anything for self-care?  Do you have a specific example? | - Overview over used methods of self-care - Preferred activity - Knowledge, when to use it - Learned by training participation? - Integration into everyday life | Do you do that regularly?  Since when do you do that?  Did you already do that before the training?  How well do you assess your personal needs?  Are there other things you do for yourself?  Do you have specific mindfulness exercises implemented into you daily life? If yes, which one? |
| **Training success**  Do you know if friends or family noticed something different about you in the last couple of weeks?  Additional: Did you notice any change regarding yourself? | - Changes noticed by relatives - Changes notices by the patient him/herself - Changes noticed by a health care professional - Why did they notice the change? | Did you notice any change regarding yourself?  Why do you think that changed?  Have you talked to one of your health care professionals about this? |
| **General self-awareness**  Self-awareness is a main topic of this mindfulness training concept: Did you notice anything new about yourself? Something you were not aware of before the training? | - Self-reflection regarding thoughts, emotions, behavior, disease symptoms - Connotation? - Comparing time before and after training participation | When did you notice this?  Is this positive or negative for you?  Why did you notice this? Does this have anything to do with your mindful practice? |
| **Body awareness (and PD symptoms)**  Can you tell me about a situation where other people pointed out that you were overestimating yourself?  Or did you notice some kind of overestimation yourself? | - Example situation - Symptoms occurred - Frequency and consequences - How often do you experience these consequences of overestimation? - Coping methods? - Additional situations? | How often do similar situations occur?  Are these situations causing interpersonal conflicts?  Did you hurt yourself because of overestimation?  Did you use a coping strategy during this situation?  Do you know other similar situations? |
| Additional question: Did you notice any change of your self-awareness of self-perception over the course of the last weeks? | - What change? - Example situation? - Any use? | Can you give me an example situation?  Is this change of any use for you?  Can you underline the use by providing an example situation? |
| **Ending: Ringing singing bowl**  Please close your eyes for a moment. You are about hear a sound. Please name your first association with this sound. | - Asking for immediate association - Reason for the association - Valency of the association - Generalization of valency in regards to mindfulness | What does this association mean for you?  Is this positive or negative for you?  Would you generalize this positive/negative association for the whole concept of mindfulness?  Can you think of any positive/negative aspects of mindfulness?  What does mindfulness mean to you? |
| Is there anything else you want to talk about? | | |
